# Vacuolar Processing Enzymes Modulating Susceptibility Response to *Fusarium oxysporum* f. sp. *cubense* Tropical Race 4 Infections in Banana

**DOI:** 10.3389/fpls.2021.769855

**Published:** 2022-01-12

**Authors:** Wan Muhamad Asrul Nizam Wan Abdullah, Noor Baity Saidi, Mohd Termizi Yusof, Chien-Yeong Wee, Hwei-San Loh, Janna Ong-Abdullah, Kok-Song Lai

**Affiliations:** ^1^Department of Cell and Molecular Biology, Faculty of Biotechnology and Biomolecular Sciences, Universiti Putra Malaysia, Serdang, Malaysia; ^2^Department of Microbiology, Faculty of Biotechnology and Biomolecular Sciences, Universiti Putra Malaysia, Serdang, Malaysia; ^3^Biotechnology and Nanotechnology Research Centre, Malaysian Agricultural Research and Development Institute, Serdang, Malaysia; ^4^Faculty of Science, School of Biosciences, The University of Nottingham Malaysia Campus, Semenyih, Malaysia; ^5^Biotechnology Research Centre, The University of Nottingham Malaysia Campus, Semenyih, Malaysia; ^6^Health Sciences Division, Abu Dhabi Women’s College, Higher Colleges of Technology, Abu Dhabi, United Arab Emirates

**Keywords:** banana, biotic stress, *Fusarium oxysporum* f. sp. *cubense*, Fusarium wilt, programmed cell death, vacuolar processing enzymes

## Abstract

*Fusarium oxysporum* f. sp. *cubense* tropical race 4 (*Foc*TR4) is a destructive necrotrophic fungal pathogen afflicting global banana production. Infection process involves the activation of programmed cell death (PCD). In this study, seven *Musa acuminata vacuolar processing enzyme* (*MaVPE1*–*MaVPE7*) genes associated with PCD were successfully identified. Phylogenetic analysis and tissue-specific expression categorized these MaVPEs into the seed and vegetative types. *Foc*TR4 infection induced the majority of *MaVPE* expressions in the susceptible cultivar “Berangan” as compared to the resistant cultivar “Jari Buaya.” Consistently, upon *Foc*TR4 infection, high caspase-1 activity was detected in the susceptible cultivar, while low level of caspase-1 activity was recorded in the resistant cultivar. Furthermore, inhibition of MaVPE activities *via* caspase-1 inhibitor in the susceptible cultivar reduced tonoplast rupture, decreased lesion formation, and enhanced stress tolerance against *Foc*TR4 infection. Additionally, the *Arabidopsis VPE*-null mutant exhibited higher tolerance to *Foc*TR4 infection, indicated by reduced sporulation rate, low levels of H_2_O_2_ content, and high levels of cell viability. Comparative proteomic profiling analysis revealed increase in the abundance of cysteine proteinase in the inoculated susceptible cultivar, as opposed to cysteine proteinase inhibitors in the resistant cultivar. In conclusion, the increase in vacuolar processing enzyme (VPE)-mediated PCD played a crucial role in modulating susceptibility response during compatible interaction, which facilitated *Foc*TR4 colonization in the host.

## Introduction

Panama disease, or Fusarium wilt, is known as the most devastating fungal disease that continuously threatens the sustainability of banana (*Musa* spp.) production on a global scale (Ploetz et al., 2015; Jamil et al., 2019). This disease is caused by the phytopathogenic soil-borne fungus known as *Fusarium oxysporum* f. sp. *cubense* (*Foc*). *Foc* tropical race 4 (*Foc*TR4) is the most virulent strain, attributable to its high compatibility with various banana cultivars (Buddenhagen, 2009). *Foc* infects bananas through small openings or wounds in the root. It then continues to proliferate in the corm and pseudostem, where it causes extensive necrosis, eventually leading to plant death (Ploetz, 2006). Several studies have shown that *Foc* exhibits a necrotrophic phase in the host plant by induction of programmed cell death (PCD) (Paul et al., 2011; Ghag et al., 2014; Dale et al., 2017). To date, two hypotheses have been proposed on how PCD is induced in *Foc*-infected banana: (1) *Foc* releases fungal toxins such as beauvericin (BEA) and fusaric acid (FA) to kill plant cells; and/or (2) re-programming of the PCD machinery in the host plant (Ploetz, 2006; Dong et al., 2019).

As an important and highly conserved process in multicellular organisms, PCD plays a central role in removing unwanted eukaryotic cells. It is involved in general cellular homeostasis, immune regulation, and developmental processes of multicellular organisms (Latrasse et al., 2016). In animal cells, activation of caspase-like proteinases has been found to be a key player in the execution of cell death (Rogers, 2006; Yu et al., 2006; Niu et al., 2013). Additionally, extensive studies on plants indicated that activation of caspase activity was required for the execution of PCD (Clarke et al., 2000; De Jong et al., 2000). However, the genes encoded for plant caspase remained a mystery until the identification of vacuolar processing enzyme (VPE) that exhibited caspase-1 activity in *Nicotiana benthamiana* (Hatsugai et al., 2004). Despite low sequence similarities between VPE and caspase-1, three-dimensional (3D) structure analysis revealed that these enzymes shared several similar enzymatic structures such as active site and substrate pockets that allowed them to cleave the substrate peptide sequence of Tyr-Val-Ala-Asp (YVAD) (Hatsugai et al., 2015). In addition, both enzymes exhibited autocatalytic conversion of the inactive precursor protein into functional proteinase (Kuroyanagi et al., 2002; Song et al., 2020).

Vacuolar processing enzyme is a cysteine proteinase legumain that belongs to the C13 proteinase family. It is responsible for processing and maturation of vacuolar proteins (Misas-Villamil et al., 2013; Vorster et al., 2019; Labudda et al., 2020). The inactive proprotein precursor of VPE is synthesized in the rough endoplasmic reticulum and activated through self-catalytic conversion in the vacuole (Hiraiwa et al., 1997; Kuroyanagi et al., 2005). An active VPE acts as an initiator to the vacuolar processing system where it activates various inactive vacuolar protein precursors, resulting in the formation of multifunctional proteins in the vacuole (Lu et al., 2016).

Plant VPE homologs are known to have pleiotropic functions that can be divided into seed- and vegetative-type isoforms (Hatsugai et al., 2004; Zhang et al., 2021). In *Arabidopsis thaliana*, four VPEs have been identified; these are known as *AtVPEα*, *AtVPEβ*, *AtVPEγ*, and *AtVPEδ* (Kinoshita et al., 1999; Gruis et al., 2002). The seed-type VPE, as exemplified by *AtVPEβ*, is essential for the processing and maturation of seed storage proteins within protein storage vacuoles (Yamada et al., 2020). The vegetative-type VPE, *AtVPEγ*, has a crucial role in plant PCD during plant defense response. For instance, upregulation of *AtVPEγ* was observed upon treatment with exogenous hormones (Kinoshita et al., 1999) and heat shock treatment (Li et al., 2012), suggesting that it played an important role in defense response against abiotic stresses.

During plant–pathogen interactions, VPE-induced PCD could progress in the form of either VPE-dependent hypersensitive response (HR) cell death or VPE-dependent susceptible cell death (Hara-Nishimura and Hatsugai, 2011). In *N. benthamiana*, silencing of *VPE* genes disrupted HR activation and promoted *turnip mosaic virus* (*TMV*) proliferation in the plant (Hatsugai et al., 2004). Likewise, *AtVPEγ* mutant lines showed increased susceptibility upon infection with *TMV* and *Botrytis cinerea* (Rojo et al., 2004). In contrast, the role of *VPE* in modulating susceptibility response was only limited to the study on mycotoxin-induced cell death in *A. thaliana* (Kuroyanagi et al., 2005). In their study, the *A. thaliana VPE*-null mutant showed increased resistance against Fumonisin-B1 (FB-1) treatment by exhibiting neither lesion formation nor vacuolar collapse in infiltrated leaves (Kuroyanagi et al., 2005). Hence, depending on the pathosystem involved, VPE could act as a dual switch by either mediating HR cell death to contain pathogen invasion or facilitating further spread of pathogen infection through VPE-dependent susceptible cell death.

Countless mitigation approaches have been implemented to avert *Foc* infection, but most of the attempts failed to provide satisfactory outcomes in field trials. To date, the only effective measure is *via* cultivation of resistant cultivars that can be obtained through genetic modification or conventional breeding. Recently, transgenic Cavendish banana overexpressing *cell death abnormality gene 9* (*Ced-9*) from *Caenorhabditis elegans*, which modulates PCD pathways, have improved tolerance against *Foc*TR4 infection (Dale et al., 2017). As such, understanding the regulation of native cell death-related genes in banana holds a great potential in unraveling the resistance mechanism during Fusarium wilt disease progression. Therefore, this study was conducted with the aim of defining the molecular characteristics of banana *VPE* genes as well as their expression during *Foc*TR4 infection in susceptible and resistant banana cultivars. In addition, we aimed to determine the underlying mechanism of banana *VPE* regulation in mediating PCD during compatible (successful disease development) and incompatible (successful plant defense) interactions with *Foc*TR4. The results could be used as a stepping-stone in generating resistant bananas in the future. In this study, we successfully characterized seven *MaVPE* genes from the banana genome of DH-Pahang (D’Hont et al., 2012), and provided valuable clues to gain insight into their specific roles as a key molecule in regulating cell death response and significantly influencing the susceptibility of *Foc*TR4-infected bananas.

## Materials and Methods

### Plant Material and Growth Conditions

In this study, two different banana varieties were used, namely, *M. acuminata* cv. Berangan (*Musa* spp. AAA group) and *M. acuminata* cv. Jari Buaya (*Musa* spp. AA group). *M. acuminata* cv. Berangan was used to demonstrate the compatible interaction (susceptible), while *M. acuminata* cv. Jari Buaya was used to demonstrate the incompatible interaction (resistant). The banana plants were grown *in vitro* and maintained in a liquid P5 medium containing a Murashige and Skoog (MS) medium (Murashige and Skoog, 1962), 2.2 mg L^–1^ 6-benzylaminopurine, 0.2 mg L^–1^ indole-3-acetic acid, 3% w/v sucrose, and 10 mg L^–1^ ascorbic acid, pH 5.8. Subculturing was performed on a monthly basis, and the plants were incubated in a 16-h light/8-h dark photoperiod at 24 ± 2°C. Wild-type (WT) *A. thaliana* (Col-0) and the *A. thaliana VPE*-null mutant were used in the loss of function analysis. T-DNA insertion lines for the *A. thaliana VPE*-null mutant were obtained from The Arabidopsis Information Resource (TAIR, germplasm identification number: CS67918). Seeds of the *A. thaliana* plant were directly sown on peat moss soil and grown in a growth room with a 16-h light/8-h dark photoperiod at 24 ± 2°C. For tissue-specific analysis, tissues samples (root, corm, pseudostem, leaves, fruit, and flowers) were collected from 10-month-old *M. acuminata* cv. Berangan. The samples collected were immediately frozen in liquid nitrogen and stored at −80°C until further use.

### *In silico* and Phylogenetic Analysis

The open reading frame (ORF) of banana and *Arabidopsis VPE*s were obtained from the Banana Genome Hub^1^ and TAIR^2^ databases, respectively. To identify the ORF sequence of MaVPEs, BLAST searches were performed in the banana genome database using all Arabidopsis VPEs as queries. Subsequently, the MaVPE protein sequences were deduced from the ORF sequence of MaVPEs. They were further examined using the CDD^3^ and PFAM^4^ databases. The molecular weight (MW) and isoelectric point (pI) of each predicted MaVPE protein sequence were calculated using ExPASy^5^. Multiple sequence alignment analysis of MaVPE proteins were performed using the ClustalW logarithm (Godo et al., 2017). Further processing of the alignment files was conducted in the BioEdit sequence alignment editor (version 7.1.3.0) with default parameter settings. Next, the phylogenetic tree of MaVPE proteins and other reported VPE protein sequences was constructed using the neighbor-joining method with the Poisson model, and bootstrap analysis was conducted using 1,000 replicates in MEGA 7.0 (Godo et al., 2017).

### Pathogen Cultivation and Inoculation

First, the *Foc*TR4 strain was grown on Komada’s agar (Komada, 1975) at 24 ± 2°C in darkness for 10 days. Conidia suspensions were then generated from the mycelium grown in Komada’s agar by incubation in sterile distilled water for 2 h and diluted to 1 × 10^6^ conidia ml^–1^. For the banana inoculation study, *M. acuminata* cv. Berangan and *M. acuminata* cv. Jari Buaya in the 7-leaf stage were inoculated for 3 h with 50 ml diluted conidia suspension and water. Then, they were transferred to a new liquid P5 medium, and the roots were harvested at the indicated times (1, 2, and 3 dpi). For the control, both cultivars were inoculated for 3 h with 50 ml sterile distilled water and harvested immediately. The root samples were collected from three biological replicates for each cultivar. All the samples were flash-frozen in liquid nitrogen and stored at −80°C until further use.

For loss of function analysis in *A. thaliana*, inoculations were performed according to a previously reported study (Edgar et al., 2006), with slight modifications. Inoculated and control WT *A. thaliana* and *A. thaliana VPE*-null mutants were gently uprooted and dipped into petri dishes containing 20 ml conidia suspension (1 × 10^6^ conidia ml^–1^) and water, respectively, for 15 min. Then, they were transplanted into a new peat moss soil. Disease severity was assessed, and the plants were photographed 0, 1, 3, 5, 7, and 10 dpi. Leaf samples were collected from three biological replicates for each plant cultivar at the indicated times. All the samples were flash-frozen in liquid nitrogen and stored at −80°C until further use.

### RNA Extraction and cDNA Synthesis

The total RNA was extracted from banana and *A. thaliana via* RNeasy Plant Mini Kit (Qiagen, Germany) according to the protocol of the manufacturer. Similarly, for tissue expression analysis, the RNA from different banana tissues were also extracted *via* RNeasy Plant Mini Kit (Qiagen, Germany) according to the protocol of the manufacturer. All the extracted RNAs were treated with EZDNase Enzyme (Thermo Fisher Scientific, United States) to remove genomic DNA contamination from the RNA. Subsequently, the integrity and quality of the RNA samples were determined by agarose gel electrophoresis and with an Implen Nanophotometer 190–1,100 nm spectrophotometer (Implen GmbH, Germany). First-strand cDNA was synthesized from 1 μg total RNA using QuantiNova Reverse Transcription Kit (Qiagen, Germany) according to the protocol of the manufacturer.

### Quantitative Real-Time Reverse Transcription PCR and Reverse Transcription PCR Analyses

The expression profile of the seven *MaVPE*s and *AtγVPE* genes in response to *Foc*TR4 infection in banana and *A. thaliana* was assessed by real-time reverse transcription PCR (RT-qPCR) analysis (Low et al., 2019). The primers used are listed in Supplementary Table 1. The 2^–ΔΔ*CT*^ method (Livak and Schmittgen, 2001) was used to determine the relative expression of the *MaVPE* and *AtVPE* genes. Transcript levels of the *MaVPE*s were normalized with two reference genes, namely, *MaGAPDH* (XM_009385982.2) and *MaUBQ2* (XM_009392617.2). *AtVPE* transcript level was normalized with two reference genes, namely, *ACT2* (NM_001338359.1) and *PEX4* (NM_001036862.2). RT-PCR was performed to assess transcript levels of the *MaVPE* genes in different tissues of *M. acuminata* cv. Berangan with four different numbers of PCR cycles (28, 32, 35, and 40 cycles). cDNA was used as a template for amplification with gene specific primers. *MaGAPDH* and *MaUBQ* were used as loading controls.

### Fungal Biomass Determination

The fungal biomass in the roots of *A. thaliana* was estimated by carrying out quantitative PCR (qPCR) (Zhang et al., 2018). Genomic DNA of *Foc*TR4 and the plant samples was extracted using NucleoSpin Plant II (Macherey-Nagel, Germany) following the instructions of the manufacturer. A total of 200 mg of the plant samples from each treatment was used for this study. The genomic DNA from the fungus was used as the template to amplify the *Foc*_242__*bp*_-specific gene alongside its corresponding specific primer set (listed in Supplementary Table 1). PCR amplification was performed using TaKara Ex Taq (Takara Biotechnology, Japan) and by following the protocol of the manufacturer. Subsequently, the amplified gene was cloned into a pGEMT-Easy vector and transformed into JM109 cells. The plasmid DNA was then isolated using NucleoSpin Plasmid (Macherey-Nagel, Germany) following the protocol of the manufacturer. The plasmid was linearized using *Eco*RV enzymes. Next, the linearized plasmid was subjected to Implen Nanophotometer 190–1,100 nm spectrophotometer (Implen GmbH, Germany) to determine its concentration and converted to copies μl^–1^ based on the following formula. Serial dilution was performed on the linearized plasmid from 10^9^ to 10^5^ copies μl^–1^. The serial dilution template was subjected to qPCR analysis to construct the standard curve. The qPCR was conducted using QuantiNova SYBR Green PCR Kit (Qiagen, Germany) and a Bio-Rad CFX 96 thermal cycler (Bio-Rad, United States) according to the protocols of the manufacturer. The amount of pathogen in the tested samples was calculated based on the results of standard curve qPCR, weight of the samples used for DNA extraction, total amount of DNA extracted, and amount of template DNA used in the qPCR reaction system. The formula used was as follows:


$$\mathrm{Copies}\,\mu \,L^{-1}=\frac{\left(6.02\times 10^{23}\,\mathrm{copies}\,{\mathrm{mol}}^{-1}\times \mathrm{concentration}\,g\,\mu \,L^{-1}\right)}{\mathrm{base}\,\mathrm{number}\times 660\,g\,m\,o\,l^{-1}}$$

### Cell Viability Assay

Cell viability was determined by measuring endogenous esterase content *via in vivo* fluorometric fluorescein diacetate (FDA) assay and histochemical microscopy analysis. The *in vivo* fluorometric FDA assay was performed according to a previous study (Watanabe and Lam, 2008). Histochemical microscopy was also conducted based on a previous study (Jones et al., 2016), with slight modifications. A dual staining solution was prepared in phosphate-buffered saline (PBS, 20 mM sodium phosphate, pH 7.4, 150 mM NaCl, 2.7 mM KCl containing 5 μg ml^–1^ FDA (Sigma-Aldrich, United States), and 10 μg ml^–1^ propidium iodide (PI) (Sigma-Aldrich, United States). Then, the root segment was hand-trimmed and stained in dual FDA/PI solution for 30 min. Next, the root segment was washed thrice with PBS and immediately observed under a ZEISS LSM 800 confocal laser microscope. Fluorescein and PI were detected using FITC and TRITC filter sets, respectively.

### Caspase Activity Assay

Relative caspase activity was determined spectrophotometrically (Rojo et al., 2004). The fluorescence value of each sample was measured using a fluorescence microplate reader (TECAN Infinite 200) with a-360 nm excitation wavelength filter and a 460-nm emission wavelength filter. Three biological replicates and three technical replicates were performed for each treatment.

### Hydrogen Peroxide (H_2_O_2_) Assay

Hydrogen peroxide assay was performed as described in a previous study (Velikova et al., 2000), with minor modifications. A total of 100 mg of root samples were homogenized into fine powder and mixed with 750 μl of 0.1% (v/v) trichloroacetic acid. Insoluble materials were removed by centrifugation at 20,000 × *g* for 10 min and 4°C. Twenty microliters of supernatant was added to 250 μl of 10 mM potassium phosphate buffer (pH 7) and 750 μl of 1 M potassium iodide before being incubated for 30 min at room temperature. Absorbance was measured at λ max = 390 nm. Hydrogen peroxide content was calculated based on hydrogen peroxide standard curve and expressed as micromolar per gram of fresh weight (μM g^–1^ FW).

### Caspase Inhibitor Analysis

Caspase inhibitor analysis was performed according to a previous report (Han et al., 2012), with slight modification. *M. acuminata* cv. Berangan plantlets in the 7-leaf stage were treated in a P5 medium containing 100 μM of caspase-1 inhibitor (Ac-YVAD-CMK) (Enzo Life, United States) for 48 h. Subsequently, the plantlets were inoculated with the *Foc*TR4 suspension (1 × 10^6^ conidia ml^–1^) for 3 h. The inoculated plantlets were then transferred to a new P5 medium and incubated in a 16-h light/8-h dark photoperiod at 24 ± 2°C for 2 days. Dimethyl sulfoxide (DMSO) was added to the medium for the control plant.

### Transmission Electron Microscopy

Root samples of banana from five different treatment groups (susceptible cultivar 0 dpi; susceptible cultivar 2 dpi; susceptible cultivar 2 dpi treated with caspase inhibitor; resistant cultivar 0 dpi, and resistant cultivar 2 dpi) were collected and subjected to both fixation and dehydration (Lai et al., 2011). Subsequently, the samples were infiltrated and embedded into beam capsules filled with a resin mixture and allowed to polymerize in an oven at 60°C for 48 h. Upon polymerization, the resin block was trimmed according to the selected area of interest. Next, ultrathin sectioning was performed using i-Ultramicrotome EM UC6 (Leica, Germany). Thin sections were stained with 4% uranyl acetate and lead citrate, and examined with a transmission electron microscope (TEM) (Leo Libra-120; Zeiss, Germany).

### Protein Extraction and Digestion

Proteomics analysis was performed on the susceptible and resistant cultivars of *M. acuminata* for two treatment groups, namely, the control group (0 dpi) and the inoculated group (2 dpi). Protein extraction from each root sample was performed similarly as described in a previous study (Wan Abdullah et al., 2021), and protein concentration was determined *via* Bradford assay (Bradford, 1976).

A total of 30 μg of protein was reconstituted in 100 μl of 50 mM ammonium bicarbonate (pH 8). In-solution digestion was performed using RapiGest (Waters Corporation, United States) and Trypsin Gold (Promega, United States) according to the protocol of the manufacturer. Tryptic digestion and RapiGest activity were terminated by the addition of 1 μl concentrated trifluoroacetic acid (TFA), followed by incubation of the samples at 37°C for 20 min. The tryptic peptide solution of each sample was centrifuged at 18,000 × *g* for 20 min. The resulting supernatants were collected and kept at −80°C until subsequent analysis.

### Nanoliquid Chromatography With Tandem Mass Spectrometry Proteomics Analysis

The protein sample was subjected to nanoliquid chromatography with tandem mass spectrometry (nanoLC-MS/MS) analysis using a Fusion Tribrid mass spectrometer (Thermo Fisher Scientific, United States) (Yang et al., 2019). For the identification of proteins, the data were searched against the UniProt *M. acuminata* database and the Amino Acid *Musa acuminata* DH Pahang v2 database^6^ with a 1% strict FDR and 5% relax FDR criteria using Percolator. Search parameters were set up according to a previous reported study (Yang et al., 2020).

### Protein Quantification and Data Analysis

Protein quantification and statistical analyses for comparative proteomic study were performed using the Perseus Software v1.6.0.7 (Max Planck Institute of Biochemistry, German) as described previously (Kok et al., 2021). Briefly, a protein file for each sample that consisted of three technical replicates in .txtformat from Proteome Discoverer was uploaded to Perseus for further comparative analysis among the samples. Proteins were considered to be significantly differentially expressed among the treatment groups with an adjusted *P*-value < 0.05.

### Statistical Analysis

All data presented were the average ± standard deviation (SD) of three biological replicates. Student’s *t*-test was performed to determine the statistically significant difference between control and inoculated plants. Significant difference was analyzed at *P* ≤ 0.05 and indicated in figure captions. The statistical analyses were conducted using the SPSS v2.0 software (IBM Corp., Armonk, NY, United States).

## Results

### Identification and Sequence Analysis of *MaVPE*s

Identification of the MaVPE genes was successfully carried out *via* homologous search in the banana genome hub database using known *VPE* sequences from *A. thaliana*, *Oryza sativa*, and *N. tabacum*. Seven *MaVPE* genes were identified and designated as *MaVPE1*, *MaVPE2*, *MaVPE3*, *MaVPE4*, *MaVPE5*, *MaVPE6*, and *MaVPE7*. Structural analysis of exons and introns of the *MaVPE*s are summarized in Supplementary Table 2. Most of the VPE homologs had similar gene structures consisting of nine exons and eight introns. However, *MaVPE3* only consisted of five exons and four introns, while *MaVPE7* contained four exons and three introns. The accession numbers of the genes and predicted polypeptide lengths, pI, and molecular masses are listed in Supplementary Table 1.

**FIGURE 1:**
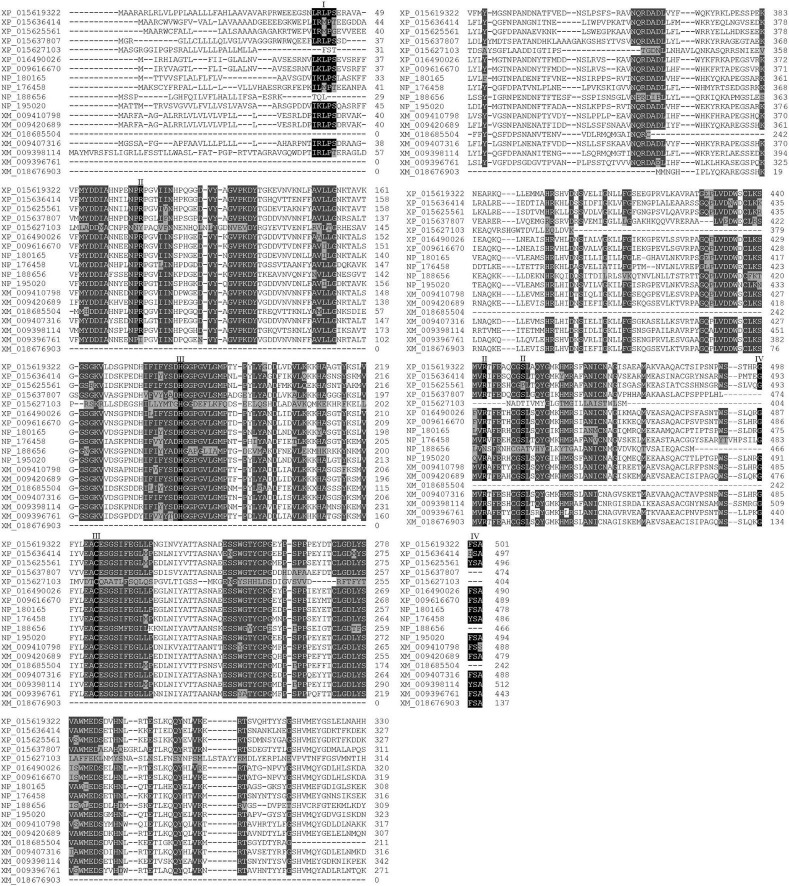
Shared and distinctive features of MaVPEs and VPEs from other plant species. Multiple sequence alignment of VPE proteins from *A. thaliana*, *O. sativa*, *N. benthamiana*, and *M. acuminata*. XP_015619322 (OsVPE1), XP_015636414 (OsVPE2), XP_015625561 (OsVPE3), XP_015637807 (OsVPE4), XP_016490026 (NbVPE1a), XP_009616670 (NbVPE1b), NP_180165 (AtαVPE), NP_176458 (AtβVPE), NP_188656 (AtδVPE), NP_195020 (AtγVPE), XP_009410798 (MaVPE1), XP_009420869 (MaVPE2), XP_018685504 (MaVPE3), XP_009407316 (MaVPE4), XP_009398114 (MaVPE5), XP_009396761 (MaVPE6), and XP_018676903 (MaVPE7). I, N-terminal vacuole signal peptide; II, ASP binding pocket; III, catalytic dyad; IV, C-terminal vacuole signal peptide.

**FIGURE 2:**
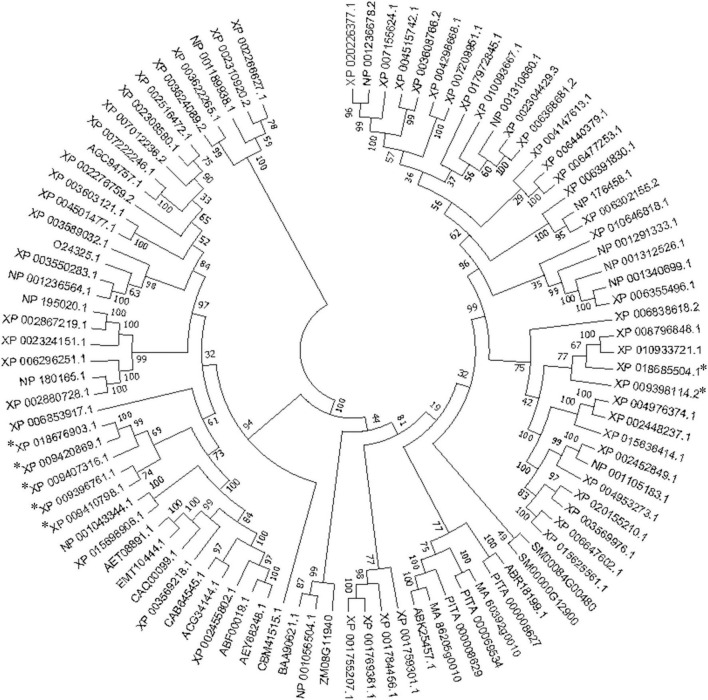
Phylogenetic relationships of vacuolar processing enzymes (VPEs) in different plant species. The phylogenetic tree was generated based on the alignment of the deduced amino acid sequence of VPE proteins from banana and other plant species. It was constructed with neighbor-joining algorithms of the MEGA 7.0 software after the multiple sequence alignment using the MUSCLE program. A bootstrap value of 1,000 replications was used to obtain support values for each branch. The MaVPE was marked with asterisk.

The deduced amino acid sequences of the seven MaVPEs revealed that MaVPE1, MaVPE2, MaVPE3, MaVPE4, MaVPE5, and MaVPE6 shared a highly conserved region of the peptidase_C13 domain. However, this domain was absent in MaVPE7. Further analysis of the amino acid sequences revealed several structural properties that were highly conserved in the VPE sequences (Figure 1 and Supplementary Figure 1). Two conserved catalytic dyad residues [His (177) and Cys (219)] were found in almost all of the MaVPE sequences. In addition, three substrate-binding pocket residues were conserved, namely, Arg (112), Arg (436), and Ser (444). The N- and C-terminal vacuole-targeting motifs represented by (I/L)(R/K)(L)(P)(S) and (G)(F/Y)(S)(A), respectively, were also identified in four MaVPEs (MaVPE1, MaVPE2, MaVPE4, and MaVPE5). Interestingly, MaVPE6 and MaVPE7 only contained a C-terminal vacuole-targeting motif. This result suggested that the MaVPEs may reside in the vacuole of the plant cell. However, no vacuole-targeting motif was observed in MaVPE3, suggesting that MaVPE3 may reside in other plant cell compartments. VPEs can be divided into seed or vegetative types depending on their functions. To group the MaVPEs, a neighbor-joining tree was created from the amino acid sequences of the seven MaVPEs and previously characterized VPEs from other plant species. As shown in Figure 2 and Supplementary Figure 2, MaVPE3 and MaVPE5are grouped together with previously identified seed-type VPEs, while the remaining five MaVPEs (MaVPE1, MaVPE2, MaVPE4, MaVPE6, and MaVPE7) are grouped into the vegetative type.

### Expression Profiles of *MaVPE*s

Insightful investigation into the transcript levels of *MaVPE*s in different plant tissues revealed distinct expression levels (Supplementary Figure 3). In general, low transcript levels of *MaVPE*s were observed in all tested plant tissues (root, corm, pseudostem, leaf, flower, and fruit), except for *MaVPE4*. For *MaVPE1* and *MaVPE2*, the highest transcript levels were observed in the root while *MaVPE4* was abundantly expressed in the fruit. On the other hand, *MaVPE3* and *MaVPE7* were similarly expressed in all the plant tissues, whereas no expression of *MaVPE5* was detected in our analysis.

**FIGURE 3:**
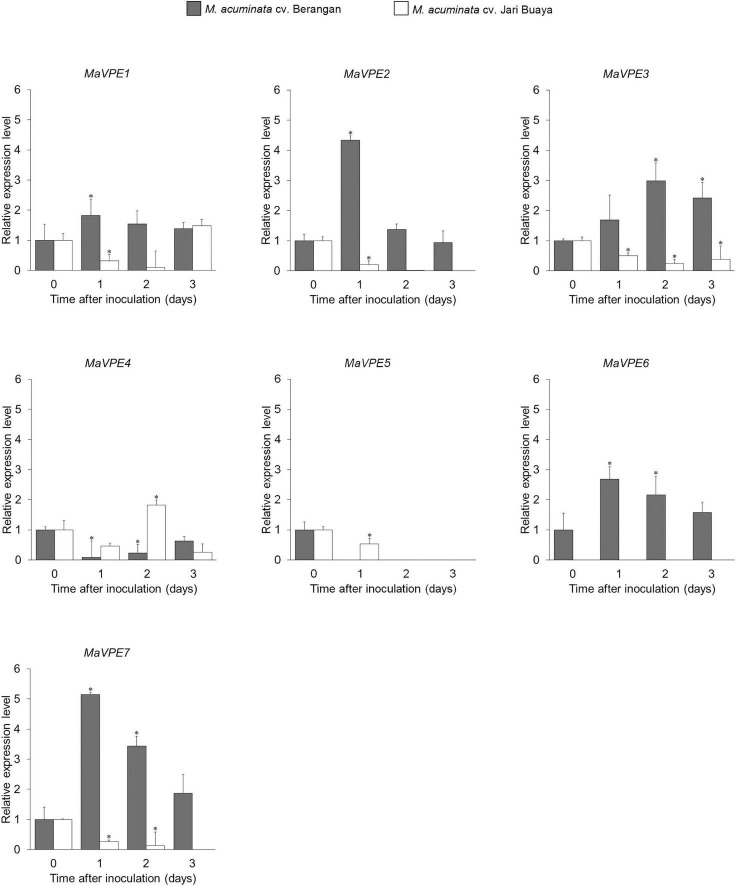
*MaVPE* gene profiling in banana roots upon *Foc*TR4 infection. Expression patterns of the *MaVPE* gene family of *Musa acuminata* cv. Berangan and *M. acuminata* cv. Jari Buaya inoculated with *Foc*TR4. Results are presented as differential relative transcript abundance. Bars represent means ± SD of three biological replicates; asterisk significantly different from control (0 dpi) at *P* ≤ 0.05 (Student’s *t*-test).

Elucidation of the role of *MaVPEs* during *Foc*TR4 infection in the roots of both susceptible and resistant banana cultivars was revealed through differentially regulated transcript levels of the seven *MaVPEs* (Figure 3). Majority of the *MaVPE* transcripts (*MaVPE1*, *MaVPE2*, *MaVPE6*, and *MaVPE7*) were induced in the susceptible cultivar with the highest expression observed 1 dpi as compared to mock-inoculated plants (0 dpi). For MaVPE3, the expression was induced starting 1 dpi and peaked 2 dpi. However, MaVPE 4 and MaVPE5 showed completely different expression patterns where they were downregulated in the susceptible cultivar. On the other hand, most of the *MaVPE* (*MaVPE2*, *MaVPE3*, *MaVPE5*, and *MaVPE7*) expressions were downregulated upon infection in the resistant cultivar. *MaVPE1* and *MaVPE4* were also found to be downregulated in the resistant cultivar in most of the time points tested. However, increments of expressions were observed 3 and 2 dpi for *MaVPE1* and *MaVPE4*, respectively. These observations indicated that the *MaVPE* transcript levels were differentially regulated and significantly affected by *Foc*TR4 infection. In general, the majority of *MaVPE*s transcript levels was induced upon infection with *Foc*TR4 during compatible interaction with the plant host, thus suggesting their important role in mediating susceptibility response.

### *Foc*TR4 Induced Cell Death During Compatible Interaction With Banana

**FIGURE 4:**
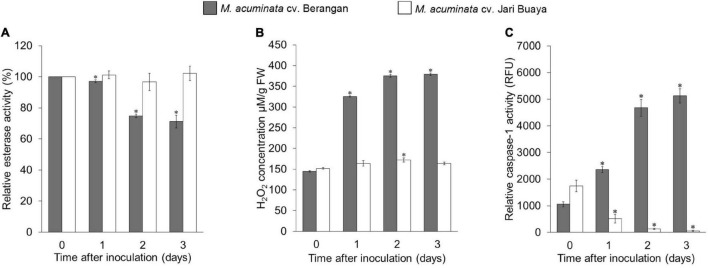
Enzymatic assays of *M. acuminata* cv. Berangan and *M. acuminata* cv. Jari Buaya inoculated with *Foc*TR4. **(A)** Relative esterase activities, **(B)** relative H_2_O_2_ levels, and **(C)** caspase-1 like activity in roots of *M. acuminata* cv. Berangan and *M. acuminata* cv. Jari Buaya inoculated with *Foc*TR4. Bars represent means ± SD of three biological replicates; asterisk significantly different from control (0 dpi) at *P* ≤ 0.05 (Student’s *t*-test).

**FIGURE 5:**
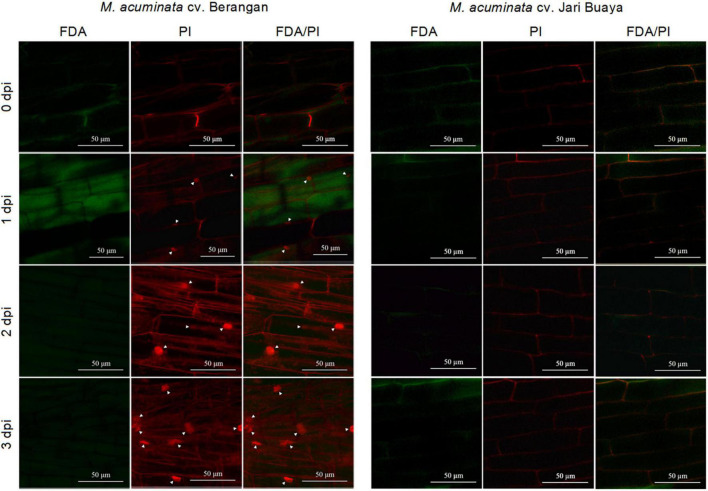
Confocal image showing the cell viability analysis based on dual staining FDA/PI. Figures show differences of cell death activity in *M. acuminata* cv. Berangan and *M. acuminata* cv. Jari Buaya upon *Foc*TR4 infection. Arrowhead indicates PI-stained nuclei.

The impact of *Foc*TR4 infection on the root system for both susceptible and resistant banana cultivars was assessed by treatment with the *Foc*TR4 suspension for up to 3 days. Our cell viability assays showed that *Foc*TR4 successfully established compatible interactions with the susceptible cultivar but not with the resistant cultivar (Figures 4A, (5). Host plant viability analysis was performed with endogenous esterase-substrate FDA (Figure 4A) and confocal microscopy analysis (Figure 5). Significant reduction in plant cell viability was observed 2 and 3 dpi in the susceptible cultivar, whereas the viability of the plant cells remained relatively unchanged at all the time points tested (Figure 4A). Similarly, a drastic loss of cell viability was exhibited in the roots of the susceptible cultivar, as shown by the *in vivo* histochemical microscopy analysis (Figure 5). Increase in cell death was observed along with increment of nucleus acid stained with PI in the susceptible cultivar infected with *Foc*TR4 (Figure 5). However, the cells of the resistant cultivar remained alive upon infection with *Foc*TR4, as shown in Figure 5. This was demonstrated by the staining of FDA and PI that remained in the cell membrane, and no nucleus acid was stained by PI. Loss of cell viability upon pathogen infection is often associated with H_2_O_2_-induced cell death. More than twofold H_2_O_2_ content was observed in the susceptible cultivar treated with *Foc*TR4 as compared to the resistant cultivar (Figure 4B). To further verify that H_2_O_2_-induced cell death in the susceptible cultivar was mediated by MaVPEs, caspase-1 activity was measured. Interestingly, MaVPE activity was observed to increase drastically 2 dpi (Figure 4C), which correlated with the induction of cell death in the susceptible plants. Taken together, these results showed that the induction of cell death during compatible interaction of *Foc*TR4 with susceptible banana was mediated by accumulation of H_2_O_2_ and increase in MaVPE activity.

### Comparative Proteomics Analysis Revealed the Signaling Cascades of MaVPE-Mediated Cell Death in *Foc*TR4-Infected Banana

Changes in the proteomes of *M. acuminata* during compatible and incompatible interactions were profiled using protein samples collected from the roots of both susceptible and resistant cultivars 0 (control) and 2 dpi (inoculated). Next, comparative proteomics analysis with the LC-MS/MS approach was carried out between the control (non-inoculated) and inoculated plantlets obtained from six independent experiments for each banana cultivar using the Perseus Software for v1.6.0.7 (Max Planck Institute of Biochemistry, German).

**FIGURE 6:**
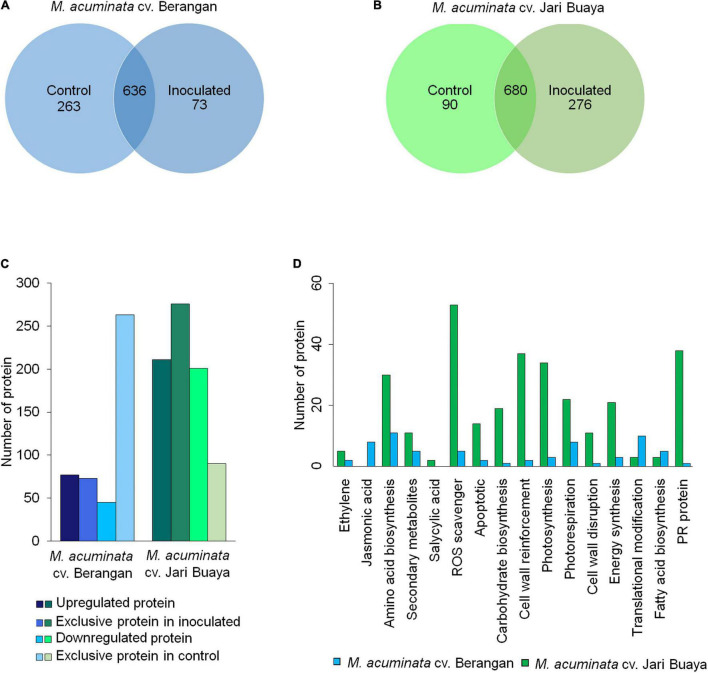
Comparative proteomic analysis of control (0 dpi) and inoculated plantlets (2 dpi) obtained from *M. acuminata* cv. Berangan and *M. acuminata* cv. Jari Buaya. Venn diagram of the total protein obtained from **(A)** *M. acuminata* cv. Berangan samples and **(B)** *M. acuminata* cv. Jari Buaya samples. **(C)** Total of differentially abundant proteins identified in both *M. acuminata* cv. Berangan samples and *M. acuminata* cv. Jari Buaya samples. **(D)** KEGG pathway analysis of differentially expressed proteins in *M. acuminata* cv. Berangan and *M. acuminata* cv. Jari Buaya.

The proteome profiles of the two treatment groups were shown to differ significantly in the Perseus analysis (Figure 6 and Supplementary Figure 4). The Pearson correlation values between the control and inoculated plants for both banana cultivars displayed high confidence values (>0.5) (Supplementary Figures 4E,F), indicating that the compared groups were derived from the same organism with minimal contamination in the samples. In total, 972 and 1,046 proteins were successfully identified in the susceptible and resistant cultivars, respectively (Figures 6A,B). In the susceptible cultivar, 263 proteins were unique to the control group, and 73 proteins were unique to the inoculated group, while 636 proteins were shared in both treatment groups. A total of 90 proteins were exclusively identified in the control group of the resistant cultivar, while 276 proteins were exclusive toward the inoculated group, and 680 proteins were found to be overlapping between both treatments. Volcanic plot analysis (Supplementary Figures 4C,D) revealed great variations in the regulation of total proteins in both banana cultivars upon *Foc*TR4 infection. In the susceptible cultivar, 77 upregulated and 45 downregulated proteins were observed after inoculation. On the other hand, in the resistant cultivar, 211 proteins were upregulated after inoculation, whereas the other 201 proteins were downregulated (Figure 6C).

Subsequently, the identified proteins were subjected to Gene Ontology (GO) analysis, by which they were categorized into three classes, namely, biological processes, molecular function, and cellular components together with total protein abundance in each group, as shown in Supplementary Figure 5. According to biological processes, majority of the proteins identified were categorized under cellular and metabolic processes for both banana cultivars. In the susceptible cultivar, 43.79 and 36.27% were identified as metabolic and cellular processes (Supplementary Figure 5A). However, in the resistant cultivar, 34.14 and 42.77% of the proteins identified were categorized as cellular and metabolic processes (Supplementary Figure 5D). Molecular function-wise, majority of the proteins identified were involved in catalytic activity and binding for both banana cultivars (Supplementary Figures 5B,E). Categorizations of the identified proteins showed that majority of the proteins were involved in binding and catalytic activities. In addition, most of the identified proteins were found to be localized at the cytoplasm and ribosomes of the cell (Supplementary Figures 5C,F).

The identified stress-related proteins were subjected to a KEGG-pathway analysis and categorized into remarkable differentially abundance protein groups (Figure 6D). As expected, significant differences were observed in the proteins associated with VPE-mediated cell death pathway, specifically in the reactive oxygen species (ROS)-scavenging proteins (Supplementary Table 3). The activation of VPE in plants upon stress induction had been found to be mediated by accumulation of ROS in many experimental systems (Li et al., 2012). In the incompatible interaction of banana and *Foc*TR4, 54 ROS-scavenging enzymes were found to be significantly increased in abundance. Meanwhile, only seven ROS-scavenging enzymes were significantly accumulated in the inoculated group of susceptible cultivar. The identified ROS-scavenging proteins included catalase, peroxidase, ferredoxin NADP^+^ reductase, and thioredoxin (Supplementary Table 3). In addition, cysteine proteinase-related protein was found to be differently regulated in both banana cultivars when inoculated with *Foc*TR4. In the susceptible cultivar, cysteine proteinase 1 was found to be exclusively expressed in the inoculated treatment group (Supplementary Table 3), which was correlated with the upregulation of *MaVPE* transcripts (Figure 3). Conversely, cysteine proteinase inhibitors 6 and 12 were upregulated in the inoculated treatment group (Supplementary Table 4), which was consistent with the expression profiling of *MaVPE*s in the resistant cultivar (Figure 3). To conclude, the results suggested that *Foc*TR4 impaired the ROS scavenging pathway. This led to ROS accumulation, which in turn activated VPE-mediated PCD in the banana plant during compatible interaction with *Foc*TR4.

### *VPE* Was Required for *Foc*TR4 *Induced-Cell Death* in *Plant*

**FIGURE 7:**
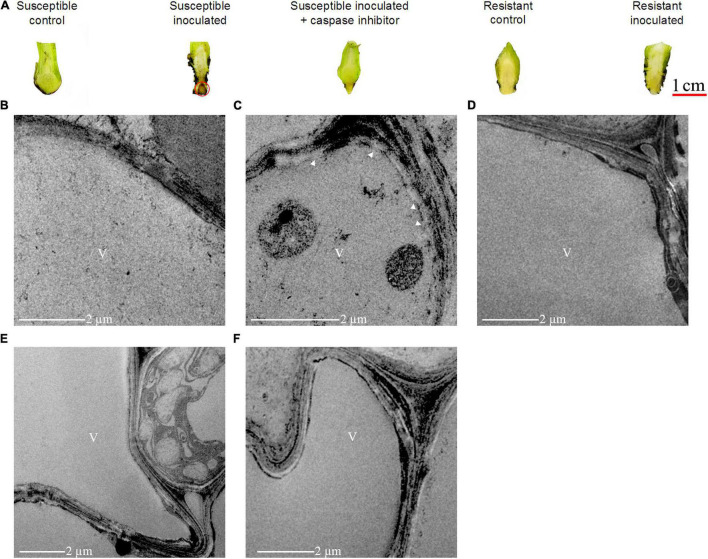
Effects of caspase-1 inhibitor on cell death induction in *Foc*TR4-infected *M. acuminata*. **(A)** Suppression of necrotic local lesion formation induced by *Foc*TR4 infection in susceptible plant treated with caspase-1 inhibitor. Caspase-1 activities were inhibited by 100 μm Ac–YVAD–CMK (caspase–1 inhibitor) for 48 h. Red circle indicates the development of necrosis. **(B–F)** Transmission electron microscopy of the root system of *M. acuminata* upon *Foc*TR4 infection. **(B)** Susceptible cultivar 0 dpi (control); **(C)** susceptible cultivar 2 dpi (inoculated); **(D)** susceptible cultivar 2 dpi (inoculated) treated with Ac-YVAD-CMK; **(E)** resistant cultivar 0 dpi (control); and **(F)** resistant cultivar 2 dpi (inoculated). White arrow indicates vacuole membrane rupture; V, vacuole.

Considering the dramatic increase of cell death 2 dpi in the susceptible cultivar (Figures 4, 5), this time point was selected to study the changes in the root morphology of both susceptible and resistant cultivars upon infection with *Foc*TR4. As shown in Figure 7A, lesion formation (labeled with red circle), a characteristic of plant cell death, was observed in the inoculated banana root of the susceptible cultivar but not in the resistant cultivar. The lesion was accompanied by disintegration of the tonoplast (Figure 7C), while the tonoplast of the resistant cultivar remained intact (Figures 7E,F). To demonstrate that VPE was required to mediate the activation of PCD in the *Foc*TR4-inoculated banana, we deployed caspase-1 inhibitor (Ac-YVAD-CMK) to inhibit MaVPE activity in the susceptible cultivar (Supplementary Figure 6). Interestingly, the inhibition of MaVPE abolished lesion formation in the roots of the susceptible inoculated banana. The vacuoles and vacuolar membranes were also found to be intact 2 dpi (Figure 7D), similar to the control group (Figure 7B).

**FIGURE 8:**
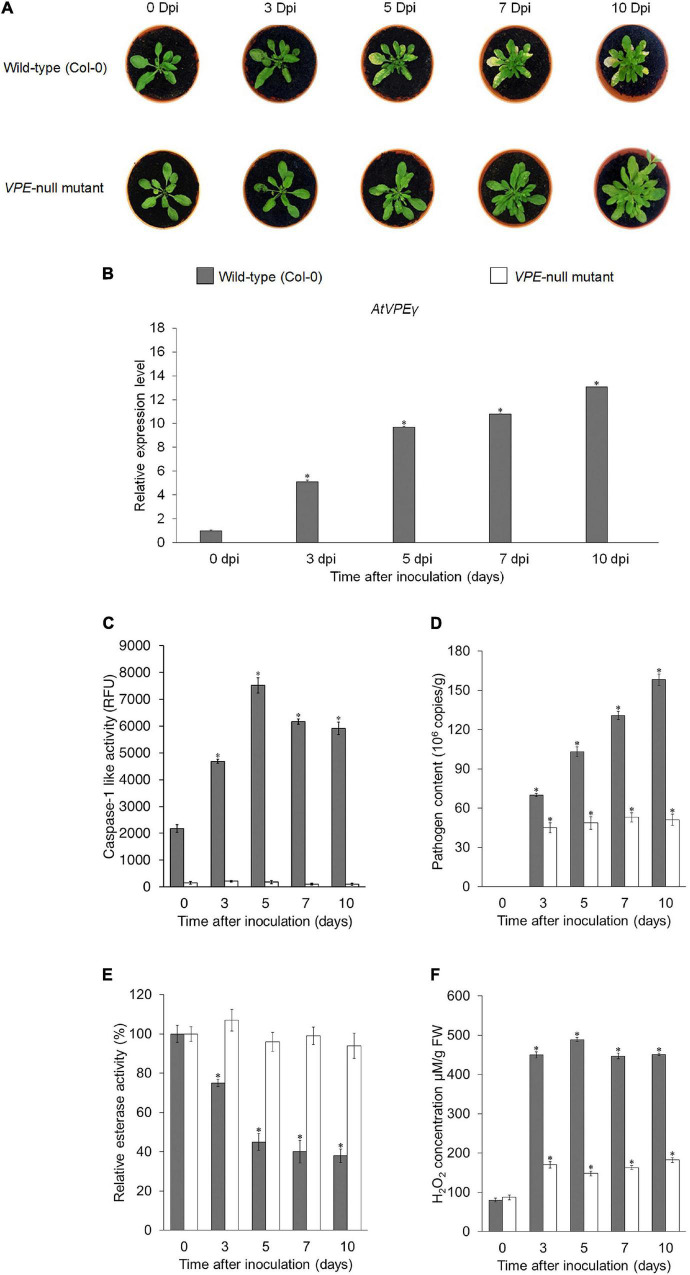
Functional analysis of *VPE* genes in *A. thaliana* upon infection with *Foc*TR4. **(A)** Disease symptoms development in *A. thaliana* wild-type (Col-0) and *A. thaliana VPE*-null mutant infected with *Foc*TR4 0, 3, 5, 7, and 10 dpi. **(B)** RT-qPCR analysis showing expression profiles of *AtγVPE* in the leaves of *Foc*TR4-inoculated *A. thaliana* wild-type (Col-0) and *A. thaliana VPE* null mutant. **(C)** Caspase-1 like activity in the leaves, **(D)** *Foc*TR4 biomass measured by qPCR in the roots, **(E)** relative esterase activities in the leaves, and **(F)** relative H_2_O_2_ content in the leaves of *A. thaliana* wild-type (Col-0) and *A. thaliana VPE* null mutant inoculated with *Foc*TR4. Bars represent means ± SD of three biological replicates; asterisk significantly different from control (0 dpi) at *P* ≤ 0.05 (Student’s *t*-test).

To further confirm the role of VPE during compatible interaction, a loss-of-function analysis was performed using the *A. thaliana VPE-*null mutant, which was deficient in all four *VPE* genes (*AtVPEα*, *AtVPEβ*, *AtVPEγ*, and *AtVPEδ*). *Foc*TR4 failed to induce lesion formation in the *A. thaliana VPE*-null mutant in the absence of functional VPE (Figure 8A). However, PCD was operational in the *A. thaliana* wild-type (Col-0) following inoculation with *Foc*TR4, as evidenced by extensive lesion formation (Figure 8A). The expression of *AtVPEγ* transcript at the onset of disease symptom (3 dpi) (Figure 8B) further verified the involvement of VPE in mediating PCD in the *Foc*TR4-inoculated host plant. However, *AtVPEα*, *AtVPEβ*, and *AtVPEδ* were not detected in all the time points tested for both the *A. thaliana* wild-type and *VPE*-null mutant (Supplementary Figure 7). These results were consistent with a previous reported study, which showed that only *AtVPEγ* was responsible for susceptibility response against mycotoxin treatment in *A. thaliana* (Kuroyanagi et al., 2005). In addition, the increase of *AtVPEγ* transcript was also supported by high levels of caspase-1 activity (Figure 8C). The activation of PCD in the inoculated *A. thaliana* wild-type (Col-0) was also accompanied by increase in *Foc*TR4 biomass (Figure 8D), increment in esterase activity (Figure 8E), and accumulation of H_2_O_2_ (Figure 8F). Taken together, these results demonstrated that VPE activity was required for the modulation of susceptibility response in *Foc*TR4-infected plants *via* the activation of PCD. The loss of VPE activity in host plants suppressed the disease symptom development, and enhanced resistance toward *Foc*TR4 infection.

## Discussion

In recent years, substantial progress had been made in understanding the role of VPE in mediating pathogen-induced PCD in plants. Clearly, the role varies according to the specific plant–pathogen being studied. “Switching-on” VPE activity in a host plant could act as a double-edged sword, because it could either benefit the plant or assist the pathogen to establish successful infection. For instance, overexpression of *SlVPE3* in tomato resulted in resistance response against *B. cinerea* (Wang et al., 2017), while inhibition of VPE activity in *A. thaliana* resulted in increased resistance toward *Hyaloperonospora arabidopsidis* infection (Misas-Villamil et al., 2013). Therefore, precise regulation of *VPE* upon pathogen infection is important in order to utilize VPE-mediated PCD as a strategy for the development of resistance in plants. In this study, it was found that *MaVPE*s, cysteine proteinases with caspase-1 activity, were involved in modulating susceptibility and resistance toward *Foc*TR4 infection in the banana plant.

### Characteristics of *MaVPE*s

Plants, like any other living organisms, require proteolytic enzymes to maintain their metabolism and physiological functions of the cells. VPEs or legumains are a type of cysteine proteinase enzyme with highly recognized functional impact on several plant physiological processes. Previously, detailed structural analyses of VPE proteins have found that VPEs shared several structural properties with caspases, including the catalytic dyad of His (174) and Cys (285) as well as a substrate binding pocket comprising of Arg (179), Arg (341), and Ser (347) (Hatsugai et al., 2006, 2015). Seven orthologs of *VPEs* in the banana genome was found; five of the banana *VPEs* (*MaVPE1*, *MaVPE2*, *MaVPE4*, *MaVPE5*, and *MaVPE6*) exhibited both His-Cys catalytic dyads and substrate-binding pockets, similar to caspase-like peptidases. The presence of conserved structures in the five MaVPEs suggests that they are able to act as cysteine proteinase enzymes. The substrate-binding pocket allows these MaVPEs to recognize specific YVAD substrates and cleave the N-termini of the substrates. Meanwhile, MaVPE3 and MaVPE7 showed a certain degree of dissimilarities as compared to the conserved regions of the VPE family. MaVPE3 lost two substrate-binding pockets, while MaVPE7 exhibited the absence of one substrate-binding pocket. Substrate-binding pockets were reported to be important for VPE to perform strong and specific binding with the caspase-1 substrate (Tang et al., 2016). Therefore, the absence of a substrate binding pocket could lead to the loss of specificity toward caspase-1 substrate. In addition, MaVPE7 also showed the absence of both His-Cys catalytic dyads, which are crucial for the proteolytic activity of VPE (Shimada et al., 1994). Although these variations may result in loss of function in MaVPE3 and MaVPE7 to catalyze the caspase-1 substrate, our data suggested that caspase-1 activity was detected in the banana plant. Therefore, it is postulated that these dissimilarities may not affect cysteine proteinase activity in the banana. Alternatively, the losses of the two MaVPEs were complemented by other MaVPEs.

Previously, phylogenetic and tissue-specific gene expression analyses allowed various VPEs to be characterized with their specific roles and classifications into seed-type VPE or vegetative-type VPE (Christoff et al., 2014; Tang et al., 2016). Based on our phylogenetic analysis, MaVPE1, MaVPE2, MaVPE4, MaVPE6, and MaVPE7 belong to the vegetative-type VPE, along with AtVPEγ and AtVPEα, which are key players in the regulation of VPE-mediated PCD in response to different biotic and abiotic stresses (Hara-Nishimura and Hatsugai, 2011; Li et al., 2012). In contrast, MaVPE3 and MaVPE5 were grouped into putative-seed type VPE. Similar seed-type VPEs such as the grape VPE (VvβVPE), *A. thaliana* VPE (AtVPEβ), and rice VPE (OsVPE1) have been reported to play important roles in the correct processing of stored proteins during seed development (Gruis et al., 2004; Wang et al., 2009; Tang et al., 2016). The results of tissue specific analyses were also concomitant with the classification obtained from the phylogenetic analysis except for *MaVPE3*, where its expression was detected in vegetative tissues even though it was categorized as seed-type VPE. Additionally, similar cases have been previously reported in seeded barley VPE (*HvLeg-2*) where it was also expressed in the leaves and showed response to both biotic and abiotic stimuli (Julián et al., 2013). Therefore, it was postulated that *MaVPE3* may hold a multifunctional role for achieving different physiological functions in both seed and vegetative tissues.

### *MaVPE* Regulation During Compatible and Incompatible Interaction of *Foc*TR4

Vacuolar processing enzyme had been identified as one of the major cysteine proteinases with reported functions in development-regulated and stress-regulated cell death in plants (Wang et al., 2009; Deng et al., 2011; Hatsugai et al., 2015). Nevertheless, no data are available on VPE from banana. Compatible interactions were defined as successful infection of the pathogen, subsequently leading to disease development in the host plant (Ponzio et al., 2016). However, incompatible interactions were explicated as successful plant defense against pathogen infection (Glazebrook, 2005). In this study, we analyzed the participation of MaVPEs in response to compatible and incompatible interactions with *Foc*TR4 infection. The results provided proof-of-principle that PCD activation in banana during the course of infection by *Foc*TR4 was regulated by the *MaVPE* genes with correlation of ROS accumulation and cell wall alteration. During the compatible interaction, *Foc* elevated the expression level of *MaVPE*s in the susceptible cultivar because of successful establishment of infection processes.

Upon attachment to the root, *Foc*TR4 was found to penetrate the epidermal cells of the root and cell walls to proliferate in the host plant (Michielse and Rep, 2009; Li et al., 2011). In our study, it was found that cell wall reinforcement proteins were regulated differently based on the interaction established between the host plant and *Foc*TR4. As shown in Supplementary Table 6, upon infection with *Foc*TR4, the cell wall reinforcement proteins increased in abundance in the resistant cultivar, but they were not found in the susceptible cultivar. In the compatible interaction, *Foc*TR4 could deploy cell wall-degrading enzymes and suppress the activation of cell wall reinforcement proteins, which facilitated further infection in the host plant. As reported previously, *Foc*TR4 secreted several CWDE such as xylanase and pectin lyase to facilitate penetration of the conidia in plant host cells (Guo et al., 2014). Both xylanase and pectin lyase could hydrolyze xylan and pectin, which are major cell wall components. As a result, *Foc*TR4 could penetrate and proliferate in host cells. Subsequently, systemic infection was present in the whole plant, and compatible interaction was established with the susceptible host plant.

In addition, CWDEs could also act as pathogen-associated molecular patterns (PAMPs), and they could be detected by a plant immunity surveillance system (Bellincampi et al., 2014; Sella et al., 2014). In the resistant cultivar, recognition of PAMPs by the plant cells could activate the signaling cascade for plant immune response. As shown in Supplementary Table 5, we observe the upregulation of pathogenesis-related (PR) proteins, which are known to be the key player in plant defense against the pathogen. PR proteins have been reported to exhibit potent antifungal properties (Ali et al., 2018). Therefore, the resistant plant could use the increased PR proteins as weapons to hydrolyze the fungal cell wall and kill the fungus. Concurrently, our results suggested that detection of *Foc*TR4 could induce cell wall enhancement proteins in the resistant plant (Supplementary Table 6). The increased cell wall enhancement proteins allowed the plant to strengthen their cellular barrier from being invaded by *Foc*TR4. It was reported that the upregulation of cell wall enhancement proteins increased the rate of the lignification process, which prevented further invasion of the pathogen in the resistant host plant (van Loon et al., 2006).

Once *Foc*TR4 penetrates the cell wall, it could impose tremendous stress in host plant cells, which could be mediated by the release of fungal toxin. Previously, studies have shown that *Foc*TR4 releases two major toxins known as BEA and FA, which contribute to the pathogenicity of the fungus (Li et al., 2013). These toxins are harmful, and the resultant stress in host cells could lead to accumulation of ROS. At the cellular level, application of both FA and BEA can destroy the banana cell and protoplast. At tissue levels, the symptoms include rotting of the roots and pseudostems, as well as wilting of the leaves (Li et al., 2013). ROS accumulation has been implicated as a subcellular messenger in multiple signaling pathways, and it could be a cause or consequence of mitochondrial alterations that eventually lead to cell death induction (Horbach et al., 2011). We inferred that the accumulation of H_2_O_2_ observed in the susceptible cultivar was the result of BEA and FA released in the host cells, which were utilized to activate the signaling cascades of VPE-mediated PCD as mentioned in a study conducted on *N. benthamiana* (Li et al., 2012). Accumulation of ROS in the plant cell was thought to activate the expression of *MaVPE*s, which led to increased VPE activity. As a result, various vacuolar proteins were activated and caused the collapse of the vacuolar membrane (Figure 6C); this could have led to the release of vacuolar contents into the cytosol. Additionally, the release of vacuolar contents such as hydrolases and vacuolar proteinases could lead to degradation and disruption of the organelle structure of the host cell and activate the caspase-1 like activity in response to *Foc*TR4 infection. Finally, host cell death during compatible interaction provides the opportunity for the pathogen to derive nutrients from the dead cell (Li et al., 2013; Ghag et al., 2014). Therefore, activation of VPE-mediated PCD facilitates *Foc*TR4-induced cell death in susceptible cultivars for efficient nutrient absorption by the pathogen.

### Inhibition of *VPE*-Mediated Programmed Cell Death Led to Enhanced Resistance to *Foc*TR4 Infection in Plant

Understanding the regulatory mechanism of PCD in plants is emerging as a promising strategy for the development of broad-spectrum resistance to abiotic and biotic stresses in plants (Li and Dickman, 2004). Such a strategy is important for generating a *Foc*TR4-resistant banana, as resources for genetic improvement are limited for this crop. Although several studies have elucidated that the inhibition of PCD could confer resistance against *Foc*TR4, the underlying mechanism remains unknown (Dickman et al., 2001; Paul et al., 2011; Dale et al., 2017). Based on our findings, this could be achieved through the inhibition of *MaVPEs* and their signaling partners.

As an obligate necrotroph, *Foc*TR4 deploys several strategies to kill the plant cell. The dead cell is then utilized as a source of nutrients, which allow it to grow, colonize, and reproduce in the plant (Ghag et al., 2014). It is postulated that this pathogen releases degradative enzymes and toxins that interact with the plant by triggering the host-cell-death pathway. Inhibition of this pathway is presumably by inhibition of VPE activity to prevent fungal infection, even though the fungus has its full complement of virulence determinants. Necrotrophic pathogens may need to co-opt with VPE-mediated PCD for successful colonization and subsequent disease development. In this study, the suppression of plant cell death observed in functional analysis using the caspase-1 inhibitor and *A. thaliana VPE*-null mutant strongly supports the notion that inhibition of VPE is the key regulator of cell death induced by *Foc*TR4. Inhibition of VPE-mediated PCD reduces cell death during the infection process and blocks the pathogen from accessing nutrients, thus reducing fungal proliferation/invasion and eventually resulting in enhanced resistance of the host plant. A similar mechanism was reported in *A. thaliana*, where the *A. thaliana VPE*-null mutant successfully inhibited susceptible-induced cell death in the host plant treated with FB-1 fungal toxin (Kuroyanagi et al., 2005). In addition, the recent development of transgenic banana overexpressing the *ced9* gene (an anti-apoptosis gene), which operates through inhibition of cell death in the plant has resulted in enhanced disease resistance to *Foc*TR4 (Ghag et al., 2014). Inhibition of cell death was proposed to improve the overall function of mitochondria and chloroplast by assisting in the generation of ATP, which is necessary for cellular homeostasis and photorespiration to prevent excessive damage by oxidative burst (Dale et al., 2017). Based on this observation, it could be concluded that the inhibition of VPE-mediated PCD enhance resistance to *Foc*TR4 infection in the plant by disrupting the nutrients released to the *Foc*TR4 or simply through maintenance of cellular homeostasis.

**FIGURE 9:**
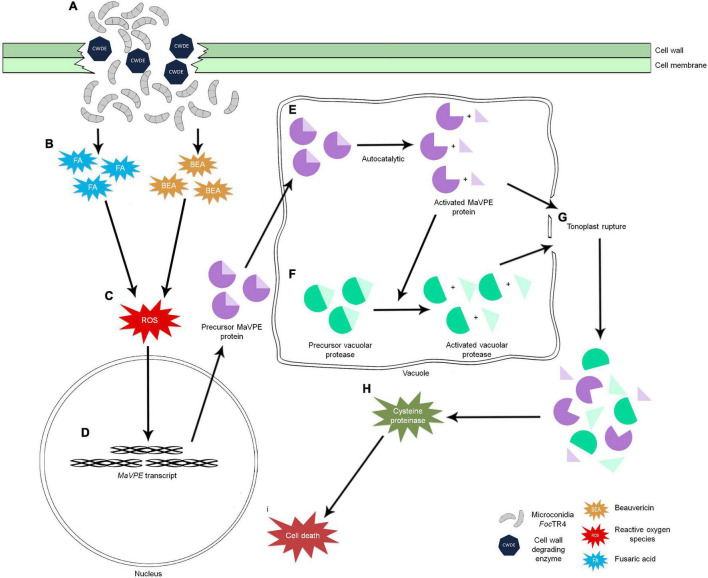
Schematic illustration of the intrinsic relationship between ROS production and regulation of VPE-mediated PCD in the plant upon *Foc*TR4 infection. **(A)** Successful penetration of *Foc*TR4 into the plant cell *via* deployment of CWDE. **(B)** *Foc*TR4 releases fungal toxins such as BEA and FA into the host plant and **(C)** triggers accumulation of ROS in host cells. ROS act as signaling molecules and **(D)** induce *MaVPE* expressions in the nucleus and translate into precursor MaVPE proteins. **(E)** Upon entering the vacuole, MaVPEs undergo an autocatalytic process to remove the N- and C-termini of the MaVPEs. **(F)** Activated MaVPEs are able to activate other vacuolar proteases and simultaneously cause the **(G)** tonoplast to rupture. Release of vacuolar proteases will **(H)** induce cysteine proteinase activity and caspase-3 like activity, **(I)** leading to cell death.

In this study, seven *MaVPE*s were successfully identified in the banana genome and systematic *in silico* analyses were performed. A series of cellular events was also revealed during *Foc*TR4-induced cell death in banana, which was associated with MaVPEs and their signaling partners. During the compatible interaction, successful *Foc*TR4 infection into the host cells was accompanied by the elevation of ROS activity, which correlates with increase in MaVPE activity (Figures 9A–F). Next, disruption of the vacuolar membrane (tonoplast) linked to the VPE activity was also observed; this could have led to the activation of cysteine proteinase activity and cell death (Figures 9G–I). It was also successfully proved that the inhibition of VPE activity through multi-gene loss of function in *A. thaliana* is crucial for the enhancement of disease resistance in the susceptible plant against *Foc*TR4 infection. Altogether, these discoveries provide new insights into the overall mechanism involved in the banana plant upon *Foc*TR4 infection specifically in VPE-mediated PCD. In the future, utilization of precise gene editing tools like CRISPR could be performed to regulate MaVPE activities in the susceptible banana plant with an aim to eventually provide better protection strategies against *Foc*TR4.

## Data Availability Statement

The original contributions presented in the study are included in the article/Supplementary Material, further inquiries can be directed to the corresponding author.

## Author Contributions

K-SL, JO-A, and WW designed this research. WW performed the research, analyzed the data, and drafted the manuscript. C-YW, H-SL, MY, and NS contributed to analytical equipment and reagents. All authors contributed to manuscript preparation.

## Conflict of Interest

The authors declare that the research was conducted in the absence of any commercial or financial relationships that could be construed as a potential conflict of interest.

## Publisher’s Note

All claims expressed in this article are solely those of the authors and do not necessarily represent those of their affiliated organizations, or those of the publisher, the editors and the reviewers. Any product that may be evaluated in this article, or claim that may be made by its manufacturer, is not guaranteed or endorsed by the publisher.

## Footnotes

1
http://banana-genome-hub.Southgreen.fr/download

2
http://www.arabidopsis.org/

3
http://www.ncbi.nlm.nih.gov/cdd/

4
http://pfam.sanger.ac.uk/

5
http://expasy.org/compute_pi/

6
https://banana-genome-hub.southgreen.fr
