# Supplementary material for: Vacuolar Processing Enzymes Modulating Susceptibility Response to Fusarium oxysporum f. sp. cubense Tropical Race 4 Infections in Banana
Source: Front Plant Sci. 2022 Jan 12;12:769855. doi: 10.3389/fpls.2021.769855 (PMC8790485; doi:10.3389/fpls.2021.769855)
Supplement: Supplementary file 1 [file Data_Sheet_1.pdf]

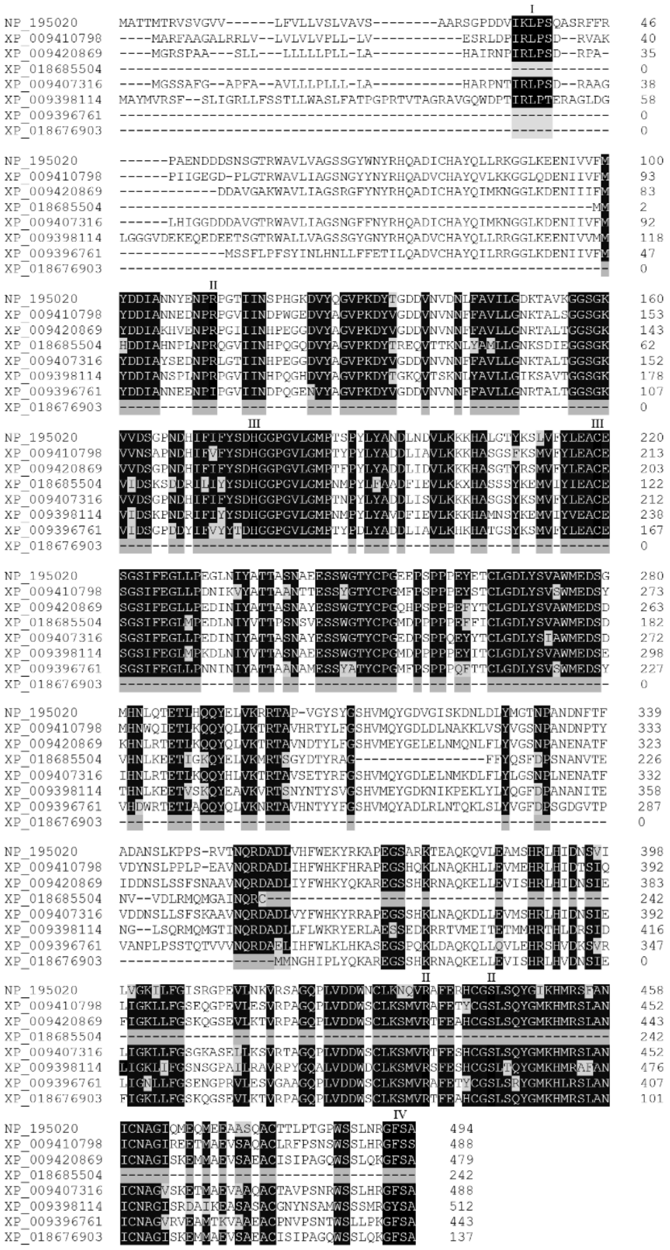

Supplementary Figure 1. Multiple sequence alignment of VPE proteins from *A. thaliana* and *M. acuminata*. NP_195020 (AtγVPE), XP_009410798 (MaVPE1), XP_009420869, (MaVPE2), XP_018685504 (MaVPE3), XP_009407316 (MaVPE4), XP_009398114 (MaVPE5), XP_009396761 (MaVPE6) and XP_018676903 (MaVPE7). I, N-terminal vacuole signal peptide; II, ASP binding pocket; III, catalytic dyad; IV, C-terminal vacuole signal peptide.

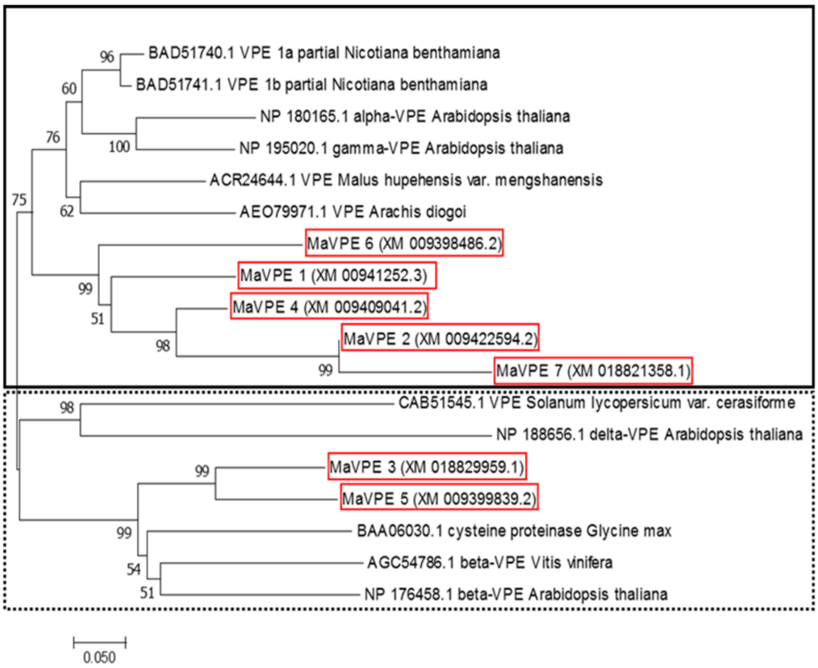

Supplementary Figure 2. Phylogenetic relationship of MaVPE with other VPE family members from different plant species. The phylogenetic tree was generated based on the alignment of the deduced amino acid sequence of VPE proteins from banana and other plant species. It was constructed by neighbour-joining algorithms of MEGA 7.0 software after the multiple sequence alignment using the MUSCLE program. A bootstrap value of 1000 replication was performed to obtain support values for each branch. Solid black box referring to vegetative VPEs, dotted black box referring to seeded VPEs and solid red boxes are VPE from banana.

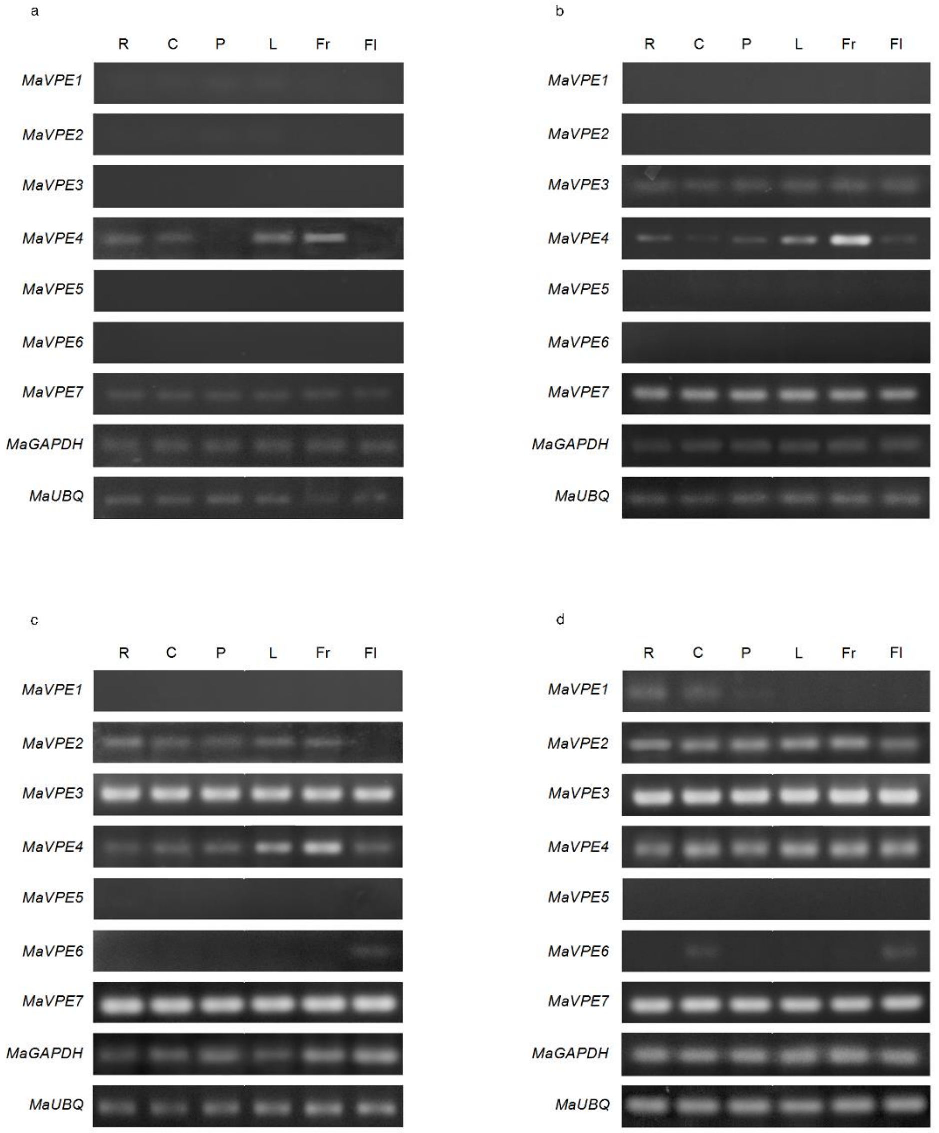

Supplementary Figure 3. Tissue-specific expression profile of *MaVPE* genes in *M. acuminata* cv. Berangan. RT-PCR analysis of tissue specific *MaVPE* genes expression 4 different number of cycles (**a**) 28 cycles, (**b**) 32 cycles, (**c**) 35 cycles and (**d**) 40 cycles**.** The total RNA was extracted from roots (R), corms (C), pseudostems (P), leaves (L), fruits (Fr) and flowers (Fl) of eight-month-old plants. *MaGAPDH* and *MaUBQ* were used as reference genes.

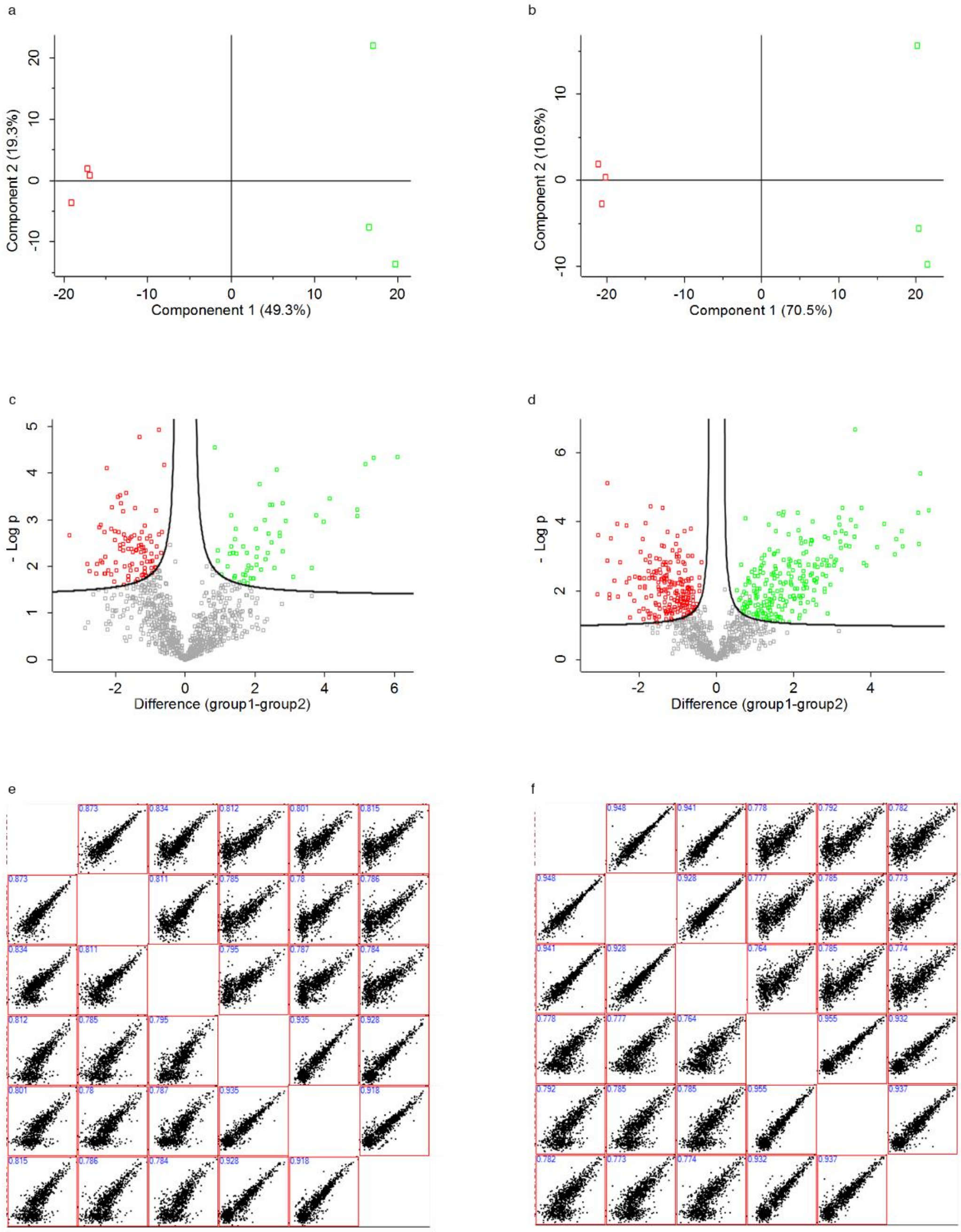

Supplementary Figure 4. Comparative proteomic analysis of control (0 dpi) and inoculated plantlets (2 dpi) obtained from *M. acuminata* cv. Berangan and *M. acuminata* cv. Jari Buaya. (**a**) Principle component analysis of control (0 dpi) (□) and inoculated (2 dpi) (□) of *M. acuminata* cv. Berangan. (**b**) Principle component analysis of control (0 dpi) (□) and inoculated (2 dpi) (□) of *M. acuminata* cv. Jari Buaya. (**c**) Volcano plot showing up- (□) and downregulated (□) proteins of the *M. acuminata* cv. Berangan samples. (**d**) Volcano plot showing up- (□) and downregulated (□) proteins of the *M. acuminata* cv. Jari Buaya samples. (**e**) Scatter plot with Pearson correlation value between biological and independent replicates of control and inoculated *M. acuminata* cv. Berangan proteome profile. (**f**) Scatter plot with Pearson correlation value between biological and independent replicates of control and inoculated *M. acuminata* cv. Jari Buaya proteome profile.

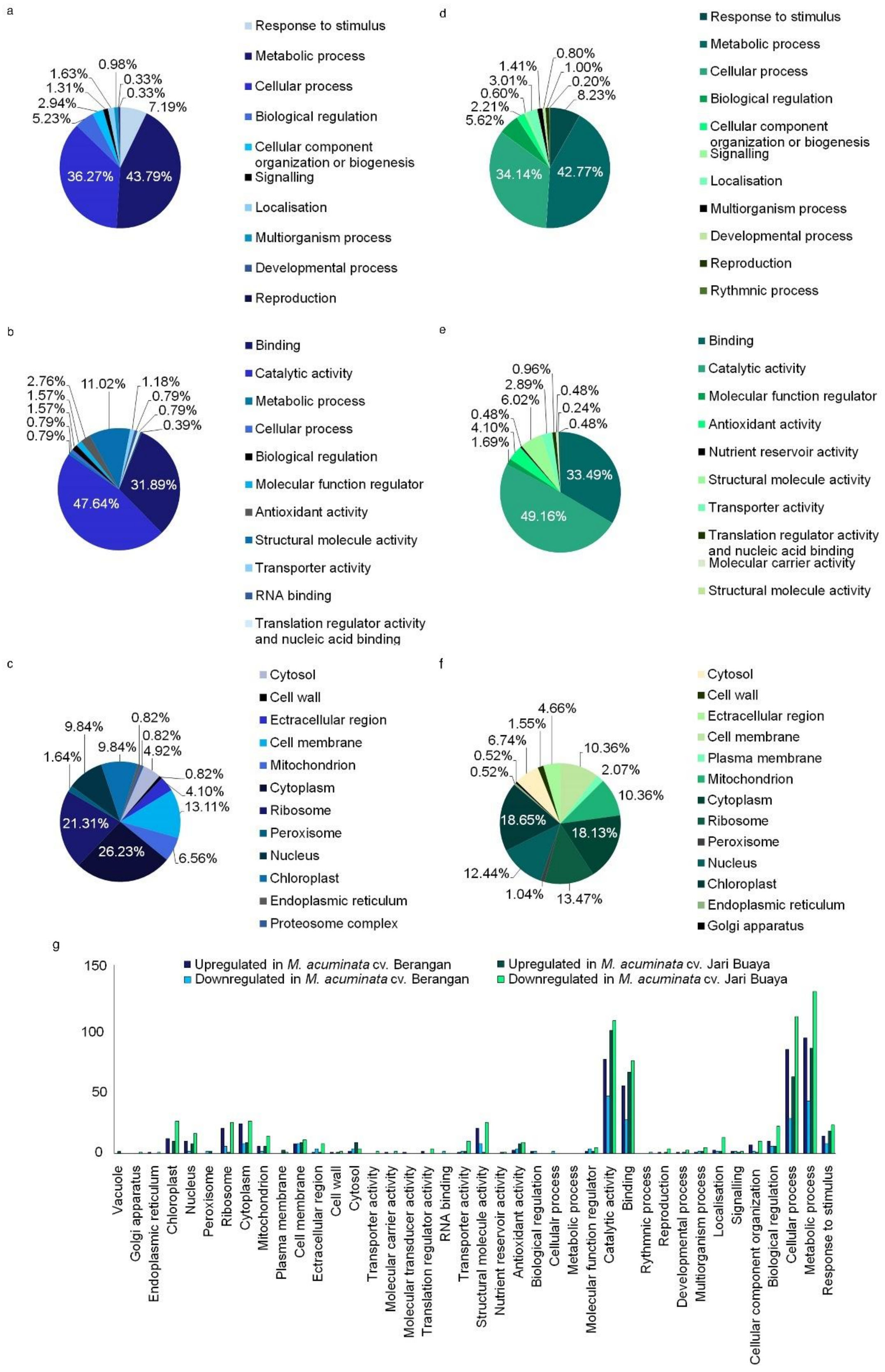

Supplementary Figure 5. Gene ontology analysis of control (0 dpi) and inoculated plantlets (2 dpi) derrived from *M. acuminata* cv. Berangan and *M. acuminata* cv. Jari Buaya proteome data. Gene ontology (GO) analysis in terms of biological processes (**a**) for *M. acuminata* cv.

Berangan samples and (**d**) *M. acuminata* cv. Jari Buaya samples. GO analysis in terms of molecular function for (**b**) *M. acuminata* cv. Berangan samples and (**e**) *M. acuminata* cv. Jari Buaya samples. GO analysis in terms of cellular components (**c**) for *M. acuminata* cv. Berangan samples and (**f**) *M. acuminata* cv. Jari Buaya samples. (**g**) Overall GO analysis in terms of biological processes, cellular components, and molecular functions, of identified proteins and their relative abundance in inoculated *M.* acuminata *cv.* Berangan and *M. acuminata* cv. Jari Buaya.

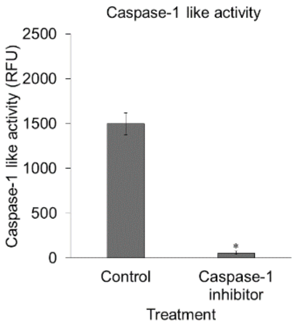

Supplementary Figure 6. Relative fluorescent activity of the susceptible *M. acuminata* cv. Berangan when treated with 100 µmoles of Ac-YVAD-CMK for 48 hours. Bars represent means ± SD of three biological replicates; * significantly different from control (0 dpi) at $P < 0.05$.

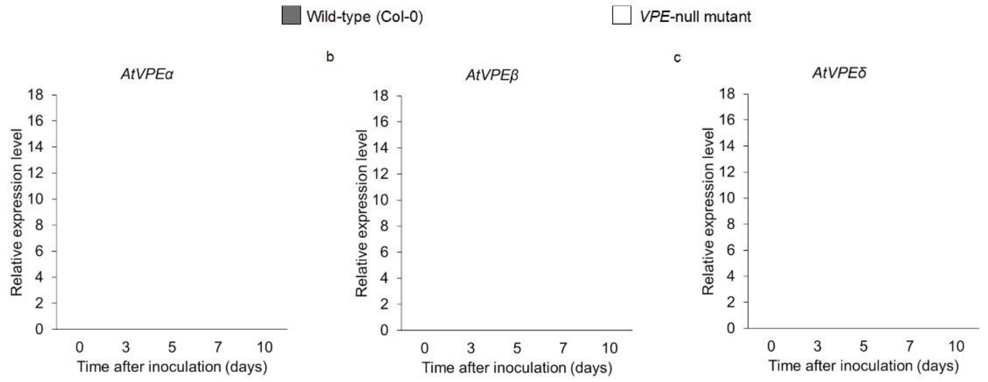

Supplementary Figure 7. Expression profile of *AtVPE*s upon infection with *Foc*TR4. RT-qPCR analysis showing expression profiles of (**a**) *AtVPE*α, (**b**) *AtVPEβ* and (**c**) *AtVPE*δ in the *Foc*TR4 inoculated *A. thaliana* wild-type (Col-0) and *A. thaliana VPE*-null mutants. Bars represent means ± SD of three biological replicates.

Supplementary Table 1. List of primer used in this study.

**Purpose**	**Primer name**	**Primer sequence**
Validation of *Foc*TR4	*TEF-1α* F	ATGGGTAAGGAAGACAAGAC
	TEF-1α R	GGAGGTACCAGTGATCATGTT
	VCG 01213 F	CACGTTTAAGGTGCCATGAGAG
	VCG 01213 R	CGCACGCCAGGACTGCCTCGTGA
Semi qRT-PCR and qRT PCR analyses in *M. acuminata*	*MaVPE1* F	CGAGCAAGGGCCTGAAGTGC
	MaVPE1 R	AGGGAGGAAACCATGGCCGA
	MaVPE2 F	ATGGGCCGATCCCCTGCC
	MaVPE2 R	CTACAACTACCGCCATCAGGCAGA
	MaVPE3 F	CACTACGAAGAACTTGTATGCTATGC
	MaVPE3 R	GTGGAAAGGTCATAGACAGCAAATCAGA
	MaVPE4 F	ATGGGCAGCTCCGCCTTC
	MaVPE4 R	GTCCAACGGATTCTTCAACTACCG
	MaVPE5 F	ATGGCATACATGGTGCGGAGCT
	MaVPE5 R	CCACGATTAGGTTGCCGACGGAG
	MaVPE6 F	TCTCGATATGGGATGAAACACCTGC
	MaVPE6 R	TCCCCAAGGGTTTCAGTGCCTGA
	MaVPE7 F	GAACGGACATATCCCATTGTATCAAAAGGC
	MaVPE7 R	GGTCAGAAGTGCTGAAAACTGTTCGC
	MaGAPDH F	CCAGCAAGGATGCCCCAATGT
	MaGAPDH R	CTGCACAACCAACTGTCTTGCT
	MaUBQ F	TCCAGCGGCTCCAAGTTCTC
	MaUBQ R	GCGAGCGTTTGGTCGTCATTC

RT-qPCR analyses in *A. thaliana*	*AtVPEα* F	TCTAGAATGACCACCGTCGTTTCCTTTCTCG
	AtVPEα R	AGATCTTCAAGCACTGAATCCAC
	AtVPEβ F	TCTAGAATGGCTAAGTCTTGCTATTTCAGAC
	AtVPEβ R	AGATCTTCAGGCGCTATAGCCTAAGAT
	AtVPEγ F	AGTGGGAAGGTTGTGGATAG
	AtVPEγ R	CTCCAGGGCAATAGGTACC
	AtVPEδ F	ATGTCTAGTCCTCTTGGTCA
	AtVPEδ R	GTTTTGCAAATCATTACATCGAACAAGCT
	ACT2 F	CTCATGCCATCCTCCGTCTT
	ACT2 R	CAATTTCCCGCTCTGCTGTT
	PEX4 F	TTACGAAGGCGGTGTTTTTC
	PEX4 R	GGCGAGGCGTGTATACATTT

Supplementary Table 2. Information of *MaVPE* gene family and their putative proteins.

Gene	Accession no.	Gene length (bp)	ORF length (bp)	Exon	Intron	Predicted		
						Size (aa)	MW (kDa)	pI
MaVPE1	XM_009412523	1467	1467	9	8	488	54229.51	5.85
MaVPE2	XM_009422594	1440	1440	9	8	479	53028.03	5.78
MaVPE3	XM_018829959	729	729	5	4	242	27079.70	4.66
MaVPE4	XM_009409041	1467	1467	9	8	488	53636.42	5.60
MaVPE5	XM_009399839	1539	1539	9	8	512	56938.49	6.10
MaVPE6	XM_009398486	1332	1332	9	8	443	48919.36	5.34
MaVPE7	XM_018821358	414	414	4	3	137	15090.42	8.98

Supplementary Table 3. List of top 20 ROS scavenging related proteins showing significant abundance difference (together with their accession numbers) in *M. acuminata* cv. Berangan and *M. acuminata* cv. Jari Buaya treated with *Foc*TR4.

No.	Accession No.	Protein Description	Protein abundance (*M. acuminata* cv. Jari Buaya)	Protein abundance (*M. acuminata* cv. Berangan)
1	Ma09_p26690.1	Oxygen-evolving enhancer protein 2-1	6.23	n/a
2	Ma01_p23600.1	Oxygen-evolving enhancer protein 1	5.61	n/a
3	M0SYW1	CBS domain-containing protein CBSX3	4.21	n/a
4	Ma05_p15800.1	Catalase isozyme 2	4.16	n/a
5	M0RGD1	Acetyltransferase component of pyruvate dehydrogenase	3.99	n/a
6	Ma08_p03860.1	Oxygen-evolving enhancer protein 2, chloroplastic	3.98	n/a
7	Ma10_p23060.2	Inosine-5'-monophosphate dehydrogenase	3.88	n/a
8	Ma04_p36490.1	Thioredoxin-like protein Clot	3.84	n/a
9	M0RPM3	Peptidyl-prolyl cis-trans isomerase CYP20-3	3.79	n/a
10	Ma08_p13920.1	Catalase isozyme A	3.70	n/a
11	M0T062	Peroxidase	3.47	n/a
12	Ma05_p22740.1	Peroxidase 5	3.42	n/a
13	D9ZHB8	Catalase (Fragment)	3.42	n/a
14	Ma11_p01370.2	Ferredoxin-NADP reductase	3.41	n/a
15	Ma07_p23680.1	Peroxidase 4	3.40	n/a
16	Ma08_p12140.1	Peroxidase 52	3.24	n/a
17	A8D5T8	Catalase	2.89	n/a
18	M0SF31	6-phosphogluconate dehydrogenase	2.82	n/a

19	Ma09_p30600.1	Putative universal stress protein A-like protein	2.79	n/a
20	Ma06_p33150.1	Thioredoxin M1, chloroplastic	2.76	n/a

n/a, not available.

Supplementary Table 4. List of cysteine proteinase proteins showing significant abundance difference (together with their accession numbers) in *M. acuminata* cv. Berangan and *M. acuminata* cv. Jari Buaya treated with *Foc*TR4.

No.	Accession No.	Protein Description	Protein abundance (*M. acuminata* cv. Jari Buaya)	Protein abundance (*M. acuminata* cv. Berangan)
1	Ma03_p17740.1	Cysteine proteinase 1	n/a	19.72*
2	Ma03_p19280.1	Cysteine proteinase inhibitor 12	1.95	n/a
3	Ma03_p31040.1	Cysteine proteinase inhibitor 6	1.50	n/a

n/a, not available
*, exclusive to *M. acuminata* cv. Berangan

Supplementary Table 5. List of top 20 PR proteins showing significant abundance difference (together with their accession numbers) in *M. acuminata* cv. Berangan and *M. acuminata* cv. Jari Buaya treated with *Foc*TR4.

No.	Accession No.	Protein Description	Protein abundance (*M. acuminata* cv. Jari Buaya)	Protein abundance (*M. acuminata* cv. Berangan)
1	Ma10_p11500.2	Pathogen-related protein	n/a	7.73
2	Q0Q293	Beta-1, 3-glucananse	5.70	n/a
3	Ma08_p22770.1	Lichenase	5.60	n/a
4	M0RY25	Endoglucanase	5.52	n/a
5	Ma01_p03480.1	Endoglucanase 17	5.11	n/a
6	Ma07_p08010.1	Pathogenesis-related protein 1	5.00	n/a
7	A0MZ69	Pathogenesis-related protein 1	4.79	n/a
8	Ma06_p19280.1	Glucanase (Fragment)	4.23	n/a
9	Ma09_p21290.1	Endoglucanase 23	3.91	n/a
10	M0RPM3	Peptidyl-prolyl cis-trans isomerase CYP20-3	3.79	n/a
11	Ma04_p03120.1	Putative Pathogenesis-related protein 1	3.54	n/a
12	Ma04_p28600.1	Thaumatin-like protein	3.05	n/a
13	Ma06_p17750.1	Tuliposide A-converting enzyme 1	2.68	n/a
14	Ma10_p06370.1	Hevamine-A	2.53	n/a
15	Ma09_p16550.1	Endochitinase EP3	2.52	n/a
16	L7TT12	1,3 beta glucanase (Fragment)	2.47	n/a
17	Ma08_p28520.1	Acidic endochitinase	2.46	n/a
18	Ma02_p08580.1	endo-1,3;1,4-beta-D-glucanase	2.08	n/a
19	Ma09_p20710.1	Endoglucanase 1	2.01	n/a
20	Ma02_p23020.1	Peptidylprolyl isomerase	1.89	n/a

n/a, not available.

Supplementary Table 6. List of top 20 cell wall reinforcement related proteins showing significant abundance difference (together with their accession numbers) in *M. acuminata* cv. Berangan and *M. acuminata* cv. Jari Buaya treated with *Foc*TR4.

**No.**	**Accession No.**	**Protein Description**	**Protein abundance (*M. acuminata* cv. Jari Buaya)**	**Protein abundance (*M. acuminata* cv. Berangan)**
1	Ma09_p15750.1	Beta-glucosidase 22	4.03	n/a
2	M0RIV9	Dirigent protein	3.92	n/a
3	Ma01_p05430.1	Guanosine nucleotide diphosphate dissociation inhibitor 2	3.80	n/a
4	Ma10_p16860.1	Putative dirigent protein 2	3.54	n/a
5	M0T062	Peroxidase	3.47	n/a
6	Ma05_p22740.1	Peroxidase 5	3.42	n/a
7	Ma07_p23680.1	Peroxidase 4	3.40	n/a
8	M0TJQ7	Peroxidase	3.40	n/a
9	Ma08_p12140.1	Peroxidase 52	3.24	n/a
10	Ma05_p31130.1	Methylesterase 3	3.21	n/a
11	Ma08_p15340.1	Guanine nucleotide-binding protein subunit beta-like protein	3.19	n/a
12	W0FAV2	Phenylalanine ammonia-lyase 1	3.03	n/a
13	Ma08_p32580.2	Putative Methylesterase 3	2.97	n/a
14	M0SBH6	Peroxidase	2.81	n/a
15	Ma10_p03220.1	Caffeoyl-CoA O-methyltransferase	n/a	2.77
16	Ma07_p07850.1	Phenylalanine ammonia-lyase	2.77	n/a
17	Ma09_p06270.1	UDP-arabinopyranose mutase 3	2.76	n/a
18	Ma06_p24120.1	Peroxidase	2.72	n/a
19	Ma08_p19730.1	Putative dirigent protein 1	2.59	n/a

20	Ma04_p02560.1	Alpha-1,4-glucan-protein synthase [UDP-forming] 2	2.48	n/a

n/a, not available.
